# BPIFA1 is a secreted biomarker of differentiating human airway epithelium

**DOI:** 10.3389/fcimb.2022.1035566

**Published:** 2022-11-28

**Authors:** Clarissa Clifton, Brian F. Niemeyer, Richard Novak, Uryan Isik Can, Kelly Hainline, Kambez H. Benam

**Affiliations:** ^1^ Division of Pulmonary, Allergy and Critical Care Medicine, Department of Medicine, University of Pittsburgh, Pittsburgh, PA, United States; ^2^ Wyss Institute for Biologically Inspired Engineering, Harvard University, Boston, MA, United States; ^3^ Division of Pulmonary Sciences and Critical Care Medicine, Department of Medicine, University of Colorado Anschutz Medical Campus, Aurora, CO, United States; ^4^ Department of Bioengineering, University of Pittsburgh, Pittsburgh, PA, United States; ^5^ Vascular Medicine Institute, University of Pittsburgh, Pittsburgh, PA, United States

**Keywords:** BPIFA1, human airway epithelium, biomarker, differentiation, inflammation

## Abstract

*In vitro* culture and differentiation of human-derived airway basal cells under air-liquid interface (ALI) into a pseudostratified mucociliated mucosal barrier has proven to be a powerful preclinical tool to study pathophysiology of respiratory epithelium. As such, identifying differentiation stage-specific biomarkers can help investigators better characterize, standardize, and validate populations of regenerating epithelial cells prior to experimentation. Here, we applied longitudinal transcriptomic analysis and observed that the pattern and the magnitude of OMG, KRT14, STC1, BPIFA1, PLA2G7, TXNIP, S100A7 expression create a unique biosignature that robustly indicates the stage of epithelial cell differentiation. We then validated our findings by quantitative hemi-nested real-time PCR from *in vitro* cultures sourced from multiple donors. In addition, we demonstrated that at protein-level secretion of BPIFA1 accurately reflects the gene expression profile, with very low quantities present at the time of ALI induction but escalating levels were detectable as the epithelial cells terminally differentiated. Moreover, we observed that increase in BPIFA1 secretion closely correlates with emergence of secretory cells and an anti-inflammatory phenotype as airway epithelial cells undergo mucociliary differentiation under air-liquid interface *in vitro*.

## Introduction

The human airway epithelium (bronchial and bronchiolar) is a pseudostratified columnar tissue that is composed of multiple epithelial cell sub-types such as basal cells, mucin-producing (goblet) cells, ciliated cells and Club cells. This mucosal barrier protects us against inhaled environmental stimuli, including pathogens, pollutants and allergens. Moreover, the airway epithelial dysfunction has been shown to be central to pathogenesis of several debilitating respiratory disorders, such as chronic obstructive pulmonary disease (COPD), asthma, primary ciliary dyskinesia (PCD), and cystic fibrosis (CF) (Martinez-Anton et al., 2013; Callejas-Diaz et al., 2020).

Over the past few decades, rigorous efforts by multiple groups led to optimization of culture conditions for *ex vivo* isolation and selective expansion, if required, of human airway epithelial cells (hAEpCs) from lung biopsies or airway brushings for subsequent *in vitro* differentiation under air-liquid interface (ALI) (Fulcher et al., 2005; Schmid et al., 2006; Ibricevic et al., 2006; Button et al., 2012; Manzanares et al., 2015; Zhao et al., 2017; Everman et al., 2018; Jiang et al., 2018). In this process, squamous-like undifferentiated monolayers of airway basal stem cells turn into terminally differentiated pseudostratified, polarized, mucociliated epithelia over the course of several weeks (typically 3–4 weeks at ALI). Such *in vitro* system is a reductionist and minimalist bottom-up approach for emulating human airway function and biology, which to a reasonable degree can reproduce tissue-level complexities of human airway epithelium. Thus, it has been widely adapted for preclinical studies into pathophysiology of lung bronchial and bronchiolar epithelia during homeostasis and when challenged with external stimuli.

Evaluating differential RNA and protein expression profiles as epithelial cells mature and differentiate has been the subject of a number of studies (Martinez-Anton et al., 2013; Callejas-Diaz et al., 2020; Bonser et al., 2021). For instance, Martinez-Anton et al., conducted global miRNA and gene expression profiling and compared day 28 (D28) well-differentiated normal human bronchial epithelial (NHBE) cells against their D0 counterpart – i.e., the undifferentiated confluent NHBE cells at time of ALI induction (Martinez-Anton et al., 2013). They found a total of 55 miRNAs and 1,201 transcript probe sets differentially expressed in D28 vs. D0. However, the authors did not characterize changes in gene expression throughout the differentiation process, as the study objective was not to elucidate biosignatures indicative of stage of hAEpCs *in vitro* differentiation. Similarly, Callejas‐Díaz and colleagues performed a transcriptome‐wide analysis on differentiating human nasal mucosa-derived basal stem cells *in vitro* (Callejas-Diaz et al., 2020). While the authors included D14 ALI samples in their studies, the focus of the study was to compare biological processes that differ during differentiation in healthy donor-derived nasal stem cells against those obtained from subjects with chronic rhinosinusitis with nasal polyps. More recently, Bonser et al., leveraging single-cell RNA-sequencing (scRNA-seq) data, devised antibody panels to characterize and isolate the major epithelial cell subsets by flow cytometry from human *in vitro* cultures (Bonser et al., 2021). Nevertheless, the overarching goal of study was not to identify differentiation stage-specific biosignatures.

Thus, we set to discover gene expression states and trajectories that correlate with various stages of mucociliary differentiation. We applied bulk transcriptomic analysis of *in vitro* cultured hAEpCs using a longitudinal sampling approach, validated our findings with sensitive hemi-nested real-time qPCR, and further confirmed one of the biomarkers by evaluating corresponding protein levels in the secretome of *in vitro* cultured hAEpCs.

## Materials and methods

### Human airway epithelial cell culture and diefferentiation

Primary human small airway and bronchial epithelial cells were either purchased from commercial sources (Promocell, Lonza, LifeLine Cell Technology) or obtained from Drs. Fernando Holguin and Oliver Eickelberg. The donor information is provided in **Supplementary Table 1**. In total, bronchial (donor *n* = 5) and bronchiolar (donor *n* = 6) cells from eleven different donors were used in our studies. Of these, eight were known non-smokers, two had smoking history, and it was unknown if one donor had any prior smoking (**Supplementary Table 1**). In addition, eight donors (who were non-smokers) were confirmed healthy by appropriate vendor/provider, two donor lungs (from subjects with smoking history) were received as healthy but we observed emphysematous changes in parts of the lung; however, cells for *in vitro* culture were isolated only from non-diseased normal areas, and for one donor (with unknow smoking history information) the cause of death was head trauma. The cells were differentiated using transwell insert (TWI) culture systems (Corning, Cat. # CLS3401). Briefly, cells were expanded in PneumaCult™ Ex-Plus media (StemCell Cat. # 0540) containing 1x hydrocortisone (StemCell Cat. # 07925) and were seeded in 24-well TWIs (0.4 µm pores; polyester membrane) at a density of 3.3e4 cells per insert (= 100,000 cells per cm^2^) and cultured until full confluence in Ex-Plus media. Then, cells were allowed to equilibrate to PneumaCult™-air-liquid interface (ALI) media (StemCell Cat. # 05001) containing 1X hydrocortisone (StemCell Cat. # 07925) and 1X heparin (StemCell Cat. # 07980) overnight followed by ALI induction through removal of apical media. Every other day basal media was changed while the cells differentiated over 28 days.

### RNA collection and whole-transcript expression analysis

Cells were lysed *in situ* for total RNA isolation on days 0, 2, 4, 6, 8, 10, 14, 21 and 28 post-ALI. For this, 200 µl of ALI media were added to the apical surface of the epithelia in TWIs and the cells were incubated for 10 minutes at 37°C. Then apical washes were performed by gently pipetting the media up and down then aspirating them to remove residual mucus and cell debris. Next, cells were lysed by adding 150 µl of RLT buffer from RNeasy Mini Kit (Qiagen Cat. # 74104). Cell lysates were then added to microcentrifuge tubes containing an additional 150 µl of the RLT buffer. RNA was isolated on-column and DNase-treated according to manufacturer’s instructions. Total RNA from the sample were then submitted to Molecular Biology Core Facilities of Dana-Farber Cancer Institute at Harvard University for analysis using Affymetrix GeneChip™ Human Gene 2.0 ST Array. The results obtained were robust multi-array average (RMA) data normalized and assessed for quality using Affymetrix Power Tools similar to our earlier microarray gene expression analysis (Benam et al., 2016), and then further processed and analyzed using custom scripts in MATLAB; duplicate genes and data lacking gene IDs were removed prior to analysis. Genes with change in expression of over two-fold over the studied time points (D0 through D28 post-ALI) and a *p* value < 0.01 (by *Kruskal-Wallis* test) were used for analysis. Data were deposited at GEO (GSE GSE197142).

### Quantitative real-time PCR analysis

RNA isolation was performed *in situ* at days 0, 2, 8, and 28 post-ALI as above. Following RNA extraction, cDNA was generated using from 5 µl of RNA template using SuperScript™ III Reverse Transcriptase (Thermo Fisher Scientific, Cat. # 18080093) and random primers (Thermo Fisher Scientific, Cat. # 4819011). Quantification gene expression was performed using hemi-nested qRT-PCR with iTaq™ Universal SYBR^®^ Green Supermix (Bio-Rad, Cat. # 1725120) using primers targeting OMG, KRT14, STC1, BPIFA1, PLA2G7, TXNIP, and S100A7 (**Supplementary Table 2**). Briefly, targeted PCR was performed using an initial set of gene specific primers for 20 cycles. The product from this PCR was then diluted 100-fold prior to a second round of PCR, consisting of 45 cycles, which utilized a new internal primer (**Supplementary Table 2**). Following this, expression levels were calculated by using the delta-delta-CT method with GAPDH serving as a reference gene, as previously described (Benam et al., 2011).

### Protein secretion analysis

Secreted protein levels were measured from the basal compartment of the TWIs at indicated days follow ALI. For BPIFA1, analysis was performed using an enzyme-linked immunosorbent assay (ELISA) kit (RayBiotech, Cat. # ELH-PLUNC-1) by following the manufacturer’s protocol. Briefly, samples and standards were loaded onto pre-coated tube strip plates and allowed to incubate at room temperature for 2 hours and 30 minutes while shaking at 150 rpm. After washing the plates, biotinylated antibody was added to the samples and they were incubated for an additional hour at room temperature at 150 rpm. After another round of washing, HRP-streptavidin was added to each well and the plates were incubated for 45 minutes at room temperature while shaking at 150 rpm. The samples were then washed and treated with 3,3,5,5’-tetramethylbenzidine solution for 30 minutes at room temperature while shaking at 150 rpm. To stop the reaction, 0.2 M sulfuric acid was added to each well. Plates were analyzed within 10 minutes of addition of the stop solution at a 450 nm wavelength using a Synergy HTX Multi-Mode Microplate Reader from BioTek on the The Gen5™ Microplate Reader and Imager software, also from BioTek.

For secreted cytokines and chemokines, the basolateral compartment media from TWIs were collected and analyzed for CSF2, IP10, MCP1, RANTES, IL6, and IL8 using custom MilliPlex assay kits (Millipore). Analyte concentrations were determined according to the manufacturer’s instructions using a Luminex FlexMap 3D system coupled with Luminex XPONENT software (Luminex).

### Flow cytometry analysis

hAEpCs at D0, 2, 8 and 35 post-ALI were removed from TWIs by incubating with Accutase (Invitrogen, Cat. #: 00-4555-56) for 20 minutes. Single-cell suspensions were fix in 4% paraformaldehyde for 15 minutes at 37C, permeabilized with 0.2% Triton X-100 for 15 minutes at room temperature, and then stained using the following primary antibodies: anti-β tubulin IV [β-tub IV] (GeneTex, Cat. #: GTX11315), anti-acetylated tubulin [α-tub] (Santa Cruz Biotechnology, Cat. #: SC-23950), anti-Keratin 5 [Krt5] (Biolegend, Cat. #: 905901), anti-nerve growth factor receptor [NGFR] (Biolegend, Cat. #: 345109), anti-mucin 5AC [Muc5AC] (Invitrogen, Cat. #: MA5-12178), anti-mucin 5B [Muc5B] (Santa Cruz Biotechnology, Cat. #: SC-21768), anti-Club cell protein 10 [CC10] (Proteintech, Cat. #: 26909-1-AP). Secondary antibodies used were Goat anti-chicken IgY-FITC (Biolegend, Cat. #: 410802), goat anti-mouse IgG-BV510 (Biolegend, Cat. #: 405331), and donkey anti-rabbit IgG-AF647(Biolegend, Cat. #: 406414). Basal cells were defined as either β-tub IV– Krt5+ or α-tub– NGFR+. Club and mucin-producing goblet cells were defined as CC10+ and Muc5AC+/Muc5B+, respectively. Ciliated cells were defined as either β-tub IV+ Krt5– or α-tub+ NGFR–.

### Statistical analysis

Statistical analysis on BPIFA1 secretion (ELISA) and flow cytometry (on frequency of epithelial sub-types) was performed by non-parametric *Mann-Whitney* test using GraphPad PRISM. The multiplexed bead-based protein levels (cytokine/chemokine secretion) were analyzed by paired Student’s *t*-test (comparing each of time points D-2, D2, D4, D6, D8, D10, D12, D14, D16, and D18 post-ALI independently against D0) using GraphPad PRISM. The data were considered statistically significant when *p* < 0.05 (*p < 0.05, **p < 0.01, ***p < 0.001, ****p < 0.0001).

## Results

**Figure 1:**
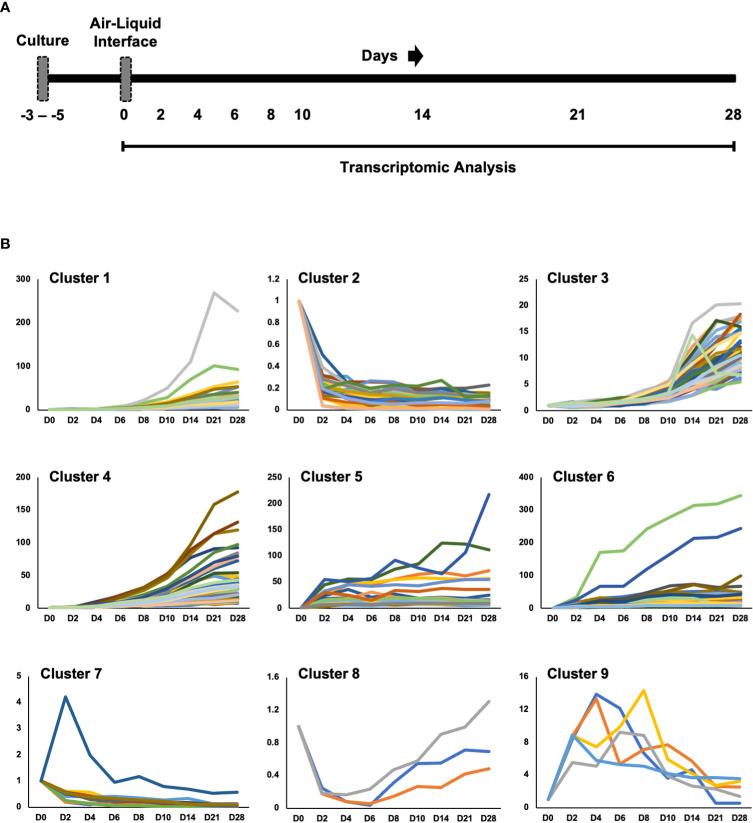
Study Design and Transcriptomic Analysis. **(A)** Diagram of healthy human small airway epithelial cells (hSAEpCs) (donor #1) guided through differentiation *in vitro* on transwell insert (TWI) porous membranes. The arrows indicate days following air-liquid interface (ALI) that cells were lysed *in situ* and used for transcriptomic analysis. **(B)** *K*-means clustering of genes differentially expressed during airway epithelial cell differentiation *in vitro* (these are genes with fold change [FC] > 8 and *p* < 0.01). D: days post-ALI; *n* = 3 replicates – i.e., TWI samples, per time point.

**Figure 2:**
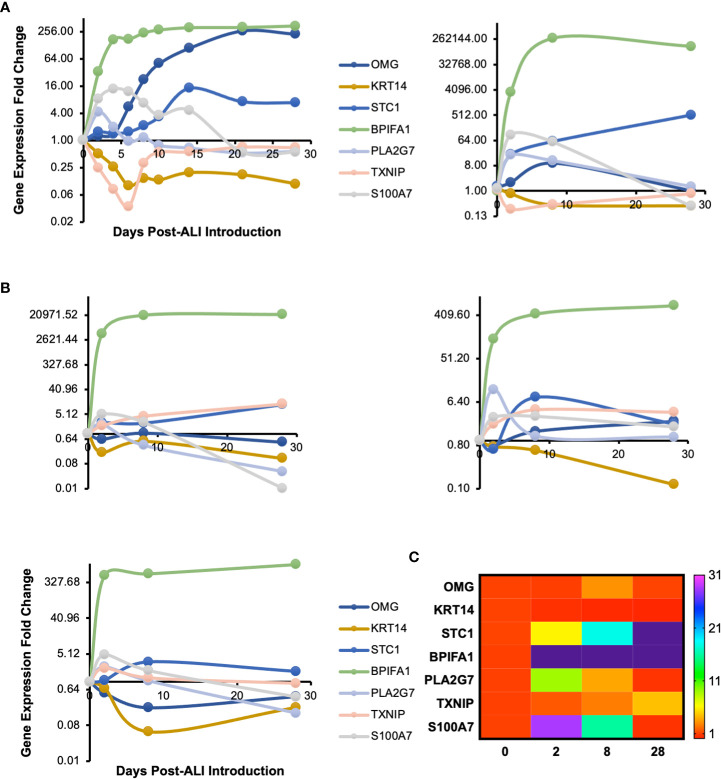
Transcriptomic Signature of *In Vitro* Differentiating Primary Human Airway Epithelial Cells. **(A)** Time course profile of expression of select genes from clusters plotted in **Figure 1B**. Left panel: microarray gene expression; right panel: hemi-nested qPCR validation of microarray data for days 0, 2, 8 and 28 post-ALI (same hSAEpC donor [donor #1]). **(B)** qPCR plots illustrating comparable patterns in expression of OMG, KRT14, STC1, BPIFA1, PLA2G7, TXNIP and S100A7 in three additional healthy human airway epithelial cell (hAEpC) donors, consistent with **(A)**. Top left (donor #2) and right (donor #3) panels: human bronchial epithelial cells [hBEpCs]: bottom left panel: hSAEpCs (donor #4). **(C)** Heatmap illustration of change in expression of genes in **(A)** and **(B)** across all tested donors (donors #1–4). OMG, oligodendrocyte myelin glycoprotein; KRT14, keratin 14; STC1, stanniocalcin 1; BPIFA1, BPI fold containing family A member 1; PLA2G7, phospholipase A2 group VII; TXNIP, thioredoxin interacting protein; S100A7, S100 calcium binding protein A7.

We cultured primary human small airway epithelial cells (hSAEpCs) *in vitro* on porous membrane of transwell inserts and guided them to full differentiation under ALI for 28 days. Then, on days 0 (that is just prior to introducing ALI), 2, 4, 6, 8, 10, 14, 21 and 28 we lysed the cells *in situ*, collected their RNA and subjected them to whole-transcript expression analysis (**Figure 1A**). Our objective here was to identify molecular signatures based on trajectories of single genes that correlate with stage of hSAEpCs differentiation. We first classified genes into similar longitudinal profiles based on similarity of their gene expression patterns for all timepoints. We detected 1,953 differentially expressed genes in total with a fold change (FC) > 2 and *p* value of < 0.01 (**Supplementary Table 3**). We further reduced the number of genes, limiting ourselves to only those with the largest magnitude change in gene expression (FC > 8 and *p* value of < 0.01), resulting in 299 identified genes of interest (**Supplementary Table 4**). Using *K*-means clustering, we fit all 299 genes into 9 clusters with distinct expression profiles in order to isolate which genes were most indicative of differentiation (**Figure 1B**). We selected the number of clusters so that the majority, if not all, of genes within each cluster demonstrate a comparable expression pattern. Next, a total of seven genes were selected (given the partial similarity in gene expression in two pairs of the clusters) as potential biomarkers having a large change in gene expression in response to stage of differentiation. These were oligodendrocyte myelin glycoprotein (OMG), keratin 14 (KRT14), stanniocalcin 1 (STC1), BPI fold containing family A member 1 (BPIFA1, also known as SPLUNC1), phospholipase A2 group VII (PLA2G7), thioredoxin interacting protein (TXNIP) and S100 calcium binding protein A7 (S100A7). To validate our findings, we performed hemi-nested real-time qPCR on these genes on four representative time points (days 0, 2, 8 and 28 following ALI). Hemi-nested real-time qPCR, as opposed to conventional qPCR, was selected to ensure that very low transcript numbers over a wide dynamic range can be reliably detected in our studies. Moreover, to address possible inter-individual variability in identified gene expression profiles, we investigated four healthy donors, encompassing bronchial and bronchiolar epithelia. As illustrated in **Figure 2A** (right panel), **2B**, the most consistent pattern of gene expression across all tested donors belonged to KRT14 and BPIFA1, both in agreement with microarray whole-transcript data [**Figures 1**, **2A** (left panel)]. The detailed changes in genes expression analysis by qPCR is shown in **Supplementary Table 5**. The expression of KRT14 fell as early as D2 post-ALI (after normalization to reference housekeeping gene, glyceraldehyde 3-phosphate dehydrogenase (GAPDH), and compared against D0) and continued to drop or stayed low until the last time-point on D28. The expression of BPIFA1 showed the exact opposite trend with a considerable increase dateable on D2, which kept on rising or stay stably high until D28. Plotting the average fold change in expression of the seven genes as heatmap (**Figure 2C**) further confirmed consistency and magnitude of change in KRT14 and BPIFA1 expression. Analysis also showed that expression of STC1, PLA2G7 and S100A7 can differentially mark D0, D2, D8 and D28 post-ALI.

**Figure 3:**
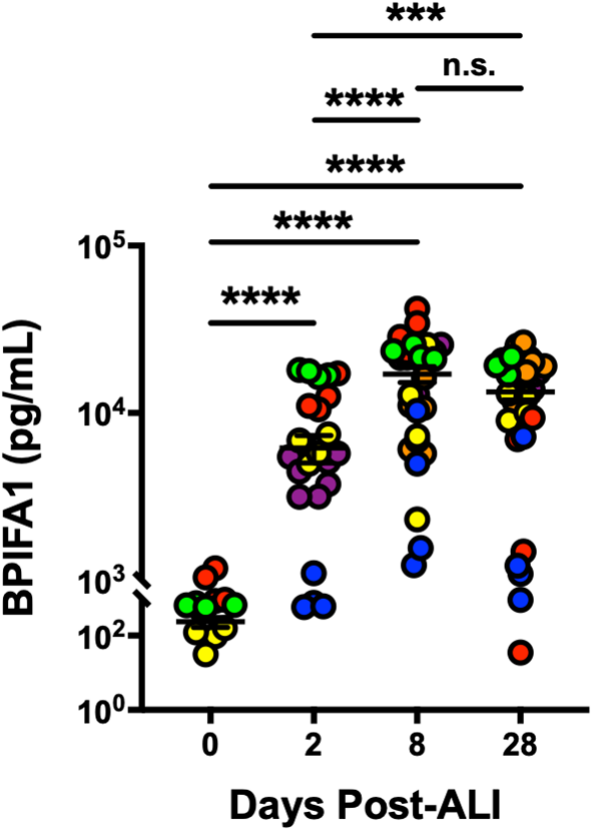
BPIFA1 Is a Secretory Biomarker of Differentiating Human Airway Epithelial Cells. Confirmation of BPIFA1 expression at protein level (secreted basolaterally into culture media) from six hAEpC donors (bronchial epithelial cells derived from two different donors [designated as yellow (donor #5) and red (donor #6)], and small airway epithelial cells derived from four separate donors [designated as purple (donor #7), orange (donor #8), blue (donor #9), and green (donor #10)]) with *n* = 4-7 replicates per donor. Data were analyzed by non-parametric *Mann-Whitney* test. ***p < 0.001, ****p < 0.0001, n.s., not significant.

BPIFA1 is an airway epithelium-derived multi-functional secretory protein that has been shown to have antimicrobial, ion channels-regulatory and airway smooth muscle-relaxing properties (Garcia-Caballero et al., 2009; Britto et al., 2013; Liu et al., 2013; Wu et al., 2017). So, we next evaluated change in secretion of BPIFA1 from basolateral surface of differentiating hAEpCs from multiple donors. We observed (**Figure 3**) that levels of BPIFA1 reached statistically significant high levels by D8 post-ALI (from undetectable at D0) and remained elevated throughout full differentiation (D28 post-ALI). This trend not only closely mimicked the transcript change in BPIFA1 expression, but also revealed that BPIFA1 can serve as a secreted biomarker of regenerating/differentiating human airway epithelium.

**Figure 4:**
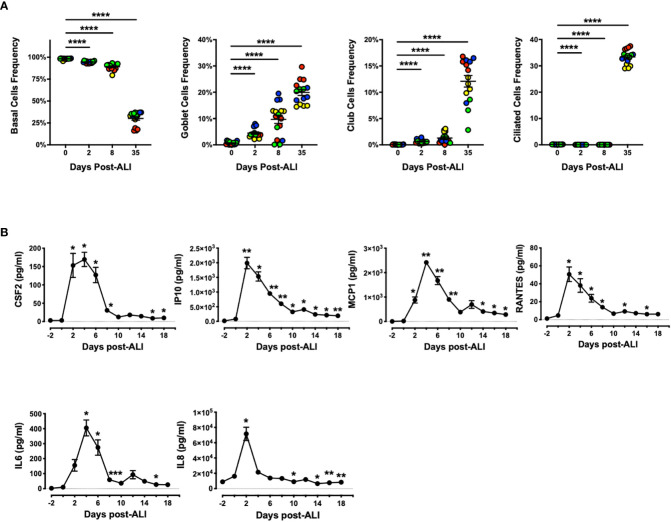
Change in Frequency of Airway Epithelial Sub-types and Secreted Inflammatory Proteins During *In Vitro* Differentiation. **(A)** The abundance of basal, goblet, Club, and ciliated cells were analyzed by flow cytometry on D0, 2, 8 and 35 post-ALI from four hAEpC donors (bronchial epithelial cells derived from two different donors [designated as yellow (donor #5) and red (donor #6)], and small airway epithelial cells derived from two separate donors [designated as blue (donor #9) and green (donor # 10)]) with *n* = 4 replicates per donor. Data were analyzed by non-parametric *Mann-Whitney* test. **(B)** Time course secretion analysis of inflammatory cytokines/chemokines for a representative human bronchial epithelial cell (donor #11) with *n* = 3–6 replicates. Data were analyzed by paired Student’s *t*-test test. *p < 0.05, **p < 0.01, ****p < 0.0001. CSF2, colony-stimulating factor 2; IP10, interferon-gamma-inducible protein 10; MCP1, monocyte chemoattractant protein-1; RANTES, regulated upon activation; normal T cell expressed and presumably secreted; IL6, interleukin 6; IL8, interleukin 8.

Next, we evaluated abundance of bronchial and bronchiolar epithelial sub-types from multiple donors as they went through ALI differentiation *in vitro* (**Figure 4A**). We observed significant increase in frequency of secretory cells (goblet and Club cells) as well as ciliated cells as the epithelial cells differentiated, while the frequency of basal cells showed the opposite trend. Notably, emergence of goblet cells occurred as early as D2 post-ALI with additional increases evident on D8 and D35, whereas marked increase in abundance of Club and ciliated cells was evident only at D35 post-ALI upon full differentiation (**Figure 4A**). Our data indicated that differentiation into mucin-producing cells shows a similar pattern to BPIFA1 transcriptomic expression and protein secretion. Lastly, we examined baseline secretion of colony-stimulating factor 2 (CSF2), interferon-gamma-inducible protein 10 (IP10), monocyte chemoattractant protein-1 (MCP1), regulated upon activation, normal T cell expressed and presumably secreted (RANTES), interleukin 6 (IL6), and IL8, as representative inflammatory cytokines and chemokines, during the course of epithelial cells differentiation (**Figure 4B**). We found that the level of all these proteins increases with induction of ALI, peaking at D2-4 post ALI before showing a downward trend to low levels. Interestingly, this pattern inversely correlates with BPIFA1 secretion.

## Discussion

In this study, we cultured and differentiated hAEpCs (bronchial and bronchiolar) *in vitro*, applied whole-transcript expression analysis and found that the profile of OMG, KRT14, STC1, BPIFA1, PLA2G7, TXNIP, S100A7 expression creates a unique biosignature that reveals the stage of epithelial cells differentiation. In addition, we successfully validated our bulk transcriptomic data in multiple donors using hemi-nested qPCR, and showed that, at protein-level, secretion of epithelium-derived pathophysiologically relevant BPIFA1 well reflects the gene expression profile. Notably the patterns of genes and protein expression were comparable between the bronchial and bronchiolar (small airway) epithelial cells. Lastly, we correlated abundance of epithelial sub-types and secretory baseline inflammatory markers with levels of BPIFA1.

Here, our overarching goal was not to delineate biological processes or pathways that are induced or inhibited upon mucociliary differentiation, rather we wanted to identify individual genes whose expression states at any given timepoint, following ALI induction and after normalization to housekeeping gene, can provide insight into hAEpC differentiation stage. To obtain meaningful results from the relatively large data set comprising of 27 samples (9 timepoints × 3 replicates per timepoint), we used *K*-means clustering for stratifying our microarray gene expression data into clusters of genes with similar longitudinal expression patterns. Since *K*-means clustering requires *a priori* knowledge of the number of clusters, we empirically found that clustering the differentially expressed genes into 9 groups yields optimal clustering to identify patterns of genes that behave similarly while avoiding multiple groups with overly similar patterns.

Our experimental strategy that led to identification of BPIFA1 as a biomarker of differentiating hAEpCs is in line with studies by Campos et al. (Campos et al., 2004), where authors detected BPIFA1 (named ‘palate, lung, nasal epithelium clone protein (PLUNC)’ at the time) as a major protein in secretions of *in vitro*-cultured hAEpCs. However, while Campos and colleagues reported a dramatic increase in BPIFA1 release into culture media of hAEpCs during the second week of ALI (consistent with our findings), their studies predominantly focused on characterization of BPIFA1 as an epithelium-derived ethanol-soluble protein. In contrast, in our studies we applied a systemic analytical approach where evaluation of whole-transcript expression data led us to identification of BPIFA1 as a biomarker of differentiating epithelial cells under ALI. Moreover, Campos et al. neither examined change in BPIFA1 mRNA expression throughout mucociliary differentiation process, nor reported a transcript signature indicative of differentiation stage.

Regulation of BPIFA1 transcript and protein levels by differentiated state of human airway epithelium provides a new angle into understanding its biological significance. The majority of prior works focused on role of immune and inflammatory signals in driving or being influenced by BPIFA1 expression (Britto et al., 2013; Britto et al., 2019; Wrennall et al., 2021). For instance, Britto and colleagues using *in vivo* murine models demonstrated that Bpifa1 is key for pulmonary recruitment and transmigration of neutrophils to the airways in response to LPS challenge, and during acute lung inflammation, the most downregulated genes in Bpifa1-/- mice are Cxcl9 and Cxcl10 (Britto et al., 2019). Similarly, Bpifa1 was downregulated in bronchoalveolar lavage fluid (BALF) of C57Bl/6 mice following viral and bacterial pneumonia, and T_h_1 and T_h_2 inflammation inhibited its protein expression *in vivo* (Britto et al., 2013). As such an insight into change in expression of BPIFA1 based on differentiated state of the airway epithelium provides a new window for further characterization of its biological significance and potentially as a prognostic biomarker.

The additional 4 genes validated using hemi-nested qPCR may find further utility in expanding the differentiation biosignature for research and clinical applications by adding additional differentiation state resolution and sensitivity. KRT14 is a biomarker of airway basal stem cells. Therefore, its expression reduction following ALI is in line with observation that the number of basal cells drops and terminally differentiated cells (e.g., ciliated cells, secretory cells) increase in frequency post-ALI induction. STC1 has been for most part studied in the setting of cellular inflammation and carcinogenesis (Yeung et al., 2012; Zhao et al., 2020); however, a recent study found that STC1 is detectable in human blood serum, its level is significantly lower in asthmatics compared with healthy control subjects, and intranasal administration of recombinant human STC1 reduces airway hyperresponsiveness and inflammation in mice (Xu et al., 2020). These findings imply a protective role for STC1 (at least in the context of allergic asthma), and our gene expression data, for the first time, reveal that STC mRNA levels increase as hAEpCs differentiate. In our studies, expression of PLA2G7 and S100A7 peaked on D2 and then started to drop to D0 or lower levels by D28 post-ALI. PLA2G7 is a secreted enzyme which degrades and inactivates platelet-activating factor (PAF) (Karabina and Ninio, 2006), yet its expression in respiratory epithelium had not been reported before. Here, we show the first evidence of PLA2G7 expression and its correlation with stage of differentiation in hAEpCs. Antimicrobial peptide S100A7, whose expression in our studies was comparable to that of PLA2G7, has been shown to be expressed by well-differentiated human airway epithelia (Andresen et al., 2011); however, here we provide the first evidence on correlation of S100A7 gene expression with stage of hAEpCs differentiation *in vitro*.

The transcriptomic molecular signature we identified in this study may serve as an initial or complementary step for utilization as diagnostic in clinical setting by detecting human airway epithelial regeneration state. This requires thorough validation of our results in large sample numbers (to be obtained, for instance, through airway brushing of healthy and diseased donors). The clinical need for such use should also be clearly laid out upfront, as airway brushing, lung biopsy acquisition or bronchoalveolar lavage fluid collection may not be feasible in all patient settings. In addition, the impact of an underlying co-morbidity must be taken into consideration.

In our studies, we also demonstrated that induction of BPIFA1 expression can likely be attributed to emergence of mucin-producing goblet cells as these cells appeared as early as two days following ALI and their number further increased on days 8 and 35. This is consistent with earlier reports. Using affinity-purified antibodies, Bingle and colleagues showed that BPIFA1 is a secreted product of goblet cells *in vitro* and in airways of whole human lungs (Bingle et al., 2010; Bingle et al., 2012). Interestingly, De Smet et al., through histochemical and transcriptomic analysis of airway in a large cohort of never-smokers and smokers with and without COPD, found that BPIFA1 is elevated in COPD patients and correlates with disease severity and goblet cell hyperplasia (De Smet et al., 2018). It has been shown that BPIFA1 expression in lungs of laboratory mice occurs in Club cells, co-localizing with secretoglobin family 1A member 1 (SCGB1A1) (Leeming et al., 2015). Thus, it is likely that this epithelial sub-type in our human *in vitro* cultures may also contribute to BPIFA1 secretion as cells differentiate.

It has been reported that BPIFA1, in addition to its antimicrobial, ion transport-influencing, and smooth muscle-relaxing properties, has an immunomodulatory function (Gaillard et al., 2010; Britto et al., 2013; Britto and Cohn, 2015; Walton et al., 2016; Wu et al., 2017; Akram et al., 2018; Khanal et al., 2021). Chu et al. reported that BPIFA1 significantly suppresses human airway epithelial IL8 production induced by *Mycoplasma pneumoniae*-derived lipoproteins and Toll-like receptor 2 (TLR2) ligand Pam3CSK4 (Chu et al., 2007). Another study reported that BPIFA1 inhibits airway eosinophilic inflammation in a murine airway allergic inflammation *in vivo*, and attenuates LPS-induced eotaxin-2 production from alveolar macrophages (Thaikoottathil et al., 2012). These findings are in line with decrease in inflammatory cytokine/chemokine secretion from hAEpCs in our studies while BPIFA1 levels go up. While we attribute the anti-inflammatory effect to BPIFA1 during epithelial cells differentiation, future studies are needed to validate this using blocking antibodies, small molecule inhibitors and/or genetic knockout systems.

Our studies here have a number of drawbacks. We drew our conclusions based on the limited number of donors we tested. In addition, we studied the differentiating epithelia in the absence of other organ-level complexities such as other lung airway cells/tissues (e.g., vascular endothelium, airway smooth muscle, fibroblasts, tissue-resident immune cells), physiologically relevant mechanical cues (e.g., rhythmic airflow associated with breathing) and sub-epithelial extracellular matrix. We also did not investigate the expression profile in diseased subject-derived epithelial cells. Future studies should address these drawbacks. Lastly, we plan in future studies to evaluate apical secretion of BPIFA1 to understand whether its levels correlate with basally secreted pattern, and to what is found in sputum or bronchoalveolar lavage of human subjects at health and disease.

In summary, this study, based on a limited number of primary human airway epithelial cell donors, reveals a unique transcriptomic molecular signature for various stages of mucociliary differentiation. It also shows that protein production of BPIFA1 closely mimics its gene expression profile, pointing to BPIFA1 as a secreted biomarker of regenerating human airway epithelium *in vitro*.

## Data availability statement

The datasets presented in this study have been deposited in Gene Expression Omnibus (GEO) under accession number GSE197142, which is available via https://www.ncbi.nlm.nih.gov/geo/query/acc.cgi?acc=GSE197142.

## Author contributions

KHB and RN conceptualized the initial gene expression studies; KHB designed and planned the study direction; KHB, CC, BFN, UC performed qPCR and protein secretion experiments; BFN performed flow cytometry studies, KHB and RN designed and planned hemi-nested qPCR; KH performed qPCR conditions optimization; RN analyzed the microarray gene expression data; KHB wrote up the manuscript. All authors contributed to the article and approved the submitted version.

## Funding

This work was supported by the Division of Pulmonary, Allergy and Critical Care Medicine at University of Pittsburgh, the Wyss Institute for Biologically Inspired Engineering at Harvard University, the U.S. National Institutes of Health (U01EB029085; R41ES031639), and the U.S. Department of Defense Congressionally Directed Medical Research Programs Discovery Award (W81XWH2010035). The views and conclusions contained in this document are those of the authors and should not be interpreted as representing the official policies, either expressed or implied, of the U.S. Department of Defense or the U.S. government.

## Acknowledgments

We thank Ms. Eva Zittel for her assistance with qPCR primers testing, staff at Molecular Biology Core Facilities, Dana-Farber Cancer Institute, for their assistance with sample preparation and microarray chip processing for gene expression studies.

## Conflict of interest

KB is founder and holds equity in Pneumax, LLC.

The remaining authors declare that the research was conducted in the absence of any commercial or financial relationships that could be construed as a potential conflict of interest.

## Publisher’s note

All claims expressed in this article are solely those of the authors and do not necessarily represent those of their affiliated organizations, or those of the publisher, the editors and the reviewers. Any product that may be evaluated in this article, or claim that may be made by its manufacturer, is not guaranteed or endorsed by the publisher.
