# Supplementary material for: BPIFA1 is a secreted biomarker of differentiating human airway epithelium
Source: Front Cell Infect Microbiol. 2022 Nov 28;12:1035566. doi: 10.3389/fcimb.2022.1035566 (PMC9744250; doi:10.3389/fcimb.2022.1035566)
Supplement: Supplementary file 6 [file Presentation_1.pdf]

# SUPPLEMENTAL INFORMATION

# BPIFA1 Is a Secreted Biomarker of Differentiating Human Airway Epithelium


## AUTHORS

Clarissa Clifton[1#], Brian F. Niemeyer[1#], Richard Novak[2#], Uryan I. Can[3#], Kelly Hainline[2], Kambez H. Benam[1,4,5*]

## AFFILIATIONS

[1]Division of Pulmonary, Allergy and Critical Care Medicine, Department of Medicine, University of Pittsburgh, Pittsburgh, PA 15213, USA. [2]Wyss Institute for Biologically Inspired Engineering, Harvard University, Boston, MA 02115, USA. [3]Division of Pulmonary Sciences and Critical Care Medicine, Department of Medicine, University of Colorado Anschutz Medical Campus, Aurora, CO 80045, USA. [4]Department of Bioengineering, University of Pittsburgh, Pittsburgh, PA 15219, USA. [5]Vascular Medicine Institute, University of Pittsburgh, Pittsburgh, PA 15213, USA. [#]These authors contributed equally.

## CONTACT

*Corresponding author: benamk@pitt.edu

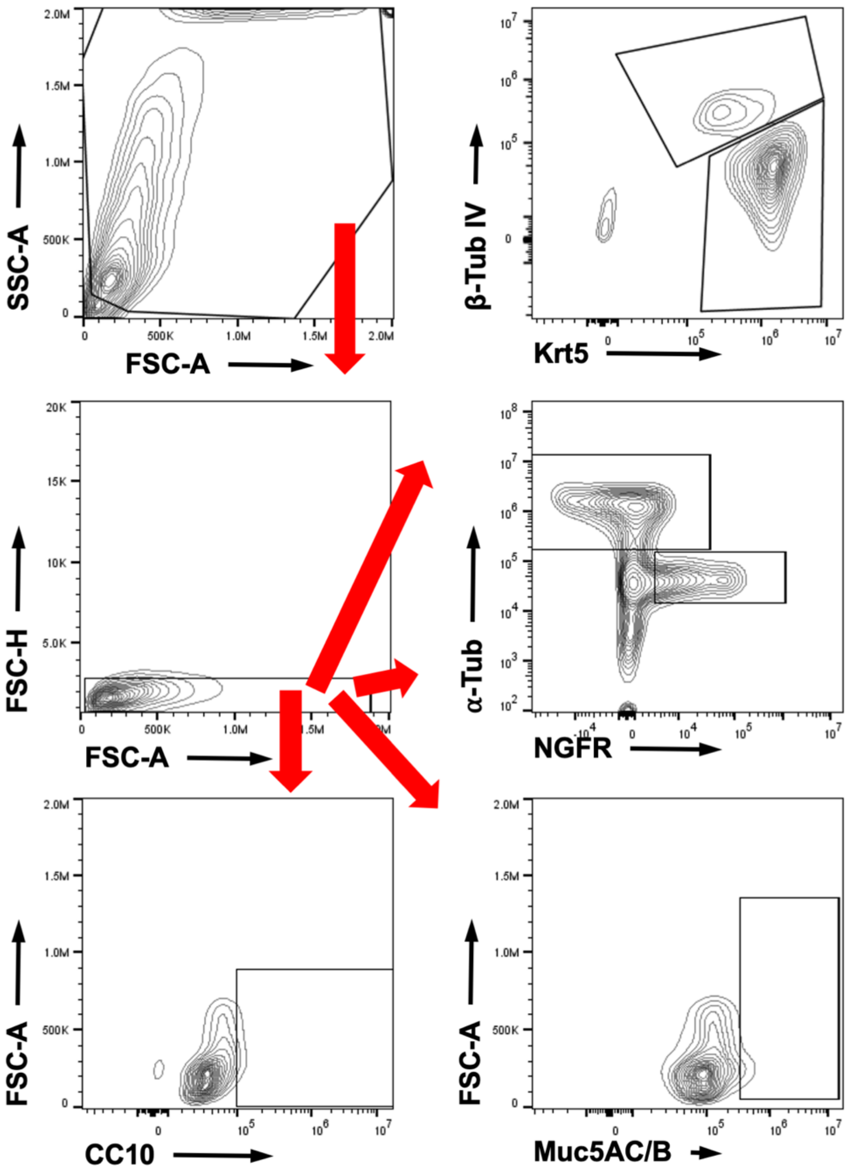

SSC-A
FSC-A
β-Tub IV
Krt5
FSC-H
FSC-A
α-Tub
NGFR
FSC-A
CC10
FSC-A
Muc5AC/B

**Supplementary Figure 1. Flow Cytometry Gating Strategies for Epithelial Cell Sub-types.** Cells were first gating using FSC-A and SSC-A parameters. Single cells were gated on using a doublet discrimination gate by FSC-A and FSC-H. From the singlet gate, basal, ciliated, Club, and goblet cells were identified. Ciliated cells were defined as either β-tubulin IV (β-tub IV) high and Keratin 5 (Krt5) negative, or Acetylated-tubulin (α-Tub) positive and Nerve Growth Factor Receptor (NGFR) negative. Basal cells were characterized as either β-tubulin IV low and $Krt5^+$ or acetylated-tubulin negative and $NGFR^+$. Club cells were defined as Club Cell 10 kDa Secretory Protein $(CC10)^+$ and goblet cells were co-stained and positive for Mucin 5AC and Mucin 5B (Muc5AC/B).

**Supplementary Table 1. Differentially Expressed Genes in Human Airway Epithelial Cells Undergoing Mucociliary Differentiation *In Vitro*.** Genes that their expression changed over 2-fold throughout differentiation with $p$ value of < 0.01 are listed here. Data is from of healthy human small airway epithelial cells (donor #1) which were guided through differentiation *in vitro* on TWI porous membranes ($n$ = 3 TWI replicate per time point).

**Supplementary Table 2. Genes with Largest Magnitude of Change During Human Airway Epithelial Cells Differentiation *In Vitro*.** Differentially expressed genes from **Supplementary Table 1**, for which change fold change (FC) was 8 is shown here.

**Supplementary Table 3. Actual Fold Change in Expression of Select Biomarker Genes for Donors Plotted in Figure 2.** ALI: Air-Liquid Interface; hSAEpC: human Small Airway Epithelial Cell; hBEpC: human Bronchial Epithelial Cell.

**Supplementary Table 4. De-identified Donor Information on Cells Used in This Study.**

**Supplementary Table 5. qPCR Primer Sequences Used in This Study.**
